# Editorial

**DOI:** 10.1120/jacmp.v1i4.2631

**Published:** 2000-09-01

With the Fall 2000 issue of the JACMP, Volume 1 is complete, and a few observations are in order.

From a modest start with four papers per issue the increase in the manuscript submission rate is such that we have been able to increase that number for this issue. If the submission rate continues to increase we will propose going to a bi‐monthly publication sometime in the near future.

There have been about 8,500 visits to the journal Web site from approximately 650 unique hosts each month with an average of 650 manuscript downloads monthly. We also know that we are reaching an international readership including Australia, Canada, China, France, Korea, etc. and we have published papers from Canada and Taiwan in addition to the USA.

Although this is an excellent start for a new journal I believe we can do better. Most of the manuscripts we receive deal with radiation oncology clinical physics. I would strongly encourage physicists working in other clinical areas such as imaging, health physics, machine maintenance and administration to submit manuscripts. I would also encourage our international readers to fully utilize the journal. We are pleased to receive manuscripts from clinical physicists worldwide.

I am very pleased to announce that three of the ACMP's Corporate Members have underwritten annual awards for the best papers published in the JACMP during the previous calendar year. They are: “The Elekta Award for Excellence” for the best professional paper, generously underwritten by Elekta, Inc.; “The LAP Award for Excellence” for the best radiation oncology physics paper, generously underwritten by LAP of America Laser App. L.C.; and “The RIT Award for Excellence” for the best paper in diagnostic physics, generously underwritten by Radiological Imaging Technology, Inc. When you have the opportunity, take a moment to thank these Corporate Members for their ongoing support of the JACMP. The initial awards will be given at next year's annual meeting.

I am also very pleased to announce that for the coming year (2001) the journal will remain subscription free. Access to and downloading of papers may be done without cost to the reader.

The successful completion of the first year of publication of the JACMP would not have been possible without the hard work of many people, including the Associate Editors and the Editorial Board, all of the referees who take time to review the manuscripts, the Manuscript Manager who keeps the whole process running smoothly, the fine people at AIP and the corporations who advertise in the journal. My thanks to all of them.

Peter R. Almond, Ph.D.

Editor‐in‐Chief

